# Decreased expression of *LRA4*, a key gene involved in rhamnose metabolism, caused up-regulated expression of the genes in this pathway and autophagy in *Pichia pastoris*

**DOI:** 10.1186/s13568-020-00971-2

**Published:** 2020-02-25

**Authors:** Jian Jiao, Shuai Wang, Hui Tian, Xinxin Xu, Yuhong Zhang, Bo Liu, Wei Zhang

**Affiliations:** Biotechnology Research Institute, Chinese Academy of Agricultural Sciences, No.12., Zhongguancun South St., Haidian District, Beijing, 100081 China

**Keywords:** *Pichia pastoris*, Rhamnose metabolism, Transcriptome, Autophagy

## Abstract

In a previous study, we developed *Pichia pastoris* GS115m, an engineered strain with decreased expression of one key gene, *LRA4*, in rhamnose metabolism. *P. pastoris* GS115m/*LacB* was subsequently constructed via introducing a β-galactosidase gene, *LacB*, under the control of rhamnose-inducible P_*LRA3*_ into *P. pastoris* GS115m. *P. pastoris* GS115m/*LacB* greatly improved recombinant protein production relative to the parental strain (*P*. *pastoris* GS115/*LacB*). In the present study, transcriptomes of *P*. *pastoris* GS115m/*LacB* and *P*. *pastoris* GS115/*LacB* grown in YPR medium were analyzed. *P*. *pastoris* GS115m/*LacB* was found to suffer from the mild carbon source starvation. To attenuate the starvation stress, *P*. *pastoris* GS115m/*LacB* attempted to enhance rhamnose metabolism by elevating the transcription levels of rhamnose-utilization genes *LRA1*-*3* and *RhaR*. The transcription level of *LacB* under the control of P_*LRA3*_ thereby increased, resulting in the improved production of recombinant protein in *P*. *pastoris* GS115m/*LacB*. It was also revealed that *P*. *pastoris* GS115m/*LacB* cells coped with carbon starvation mostly via autophagy.

## Introduction

Because of its toxic and flammable properties, the use of methanol is dangerous during recombinant protein production. Hence, safe compound-inducible promoters are needed as alternatives to the methanol-inducible promoter P_*AOX1*_ in *Pichia pastoris* expression systems (Vogl and Glieder 2013). Several strong and inducible promoters, such as P_*PHO89*_ or P_*THI11*_, have been discovered to date (Ahn et al. 2009; Stadlmayr et al. 2010). Extensive efforts have been made in our laboratory to identify some other alternatives to P_*AOX1*_, and we have discovered several rhamnose utilization related genes (four enzyme-coding genes, *LRA1*–*4*, and one regulator-coding gene, *RhaR*). Simultaneously, the promoters of *LRA3* and *LRA4*, P_*LRA3*_ and P_*LRA4*_, were identified as two strong rhamnose-inducible promoters (Jiao et al. 2019; Liu et al. 2016). Subsequently, a *Pichia* expression system based on P_*LRA3*_, designated as the P_*LRA3*_ system, was developed using rhamnose as the inducer. However, the P_*LRA3*_ system did not produce recombinant proteins as efficiently as the P_*AOX1*_ system. To enhance recombinant protein production in the P_*LRA3*_ system, the engineered strain *P. pastoris* GS115m with decreased rhamnose metabolism flux was constructed via replacing the strong rhamnose-inducible promoter P_*LRA4*_ by another weak rhamnose-inducible promoter P_*LRA2*_. *P. pastoris* GS115m presented several different profiles compared to the parental strain *P. pastoris* GS115 as follows: (i) lower rhamnose utilization rate due to decreased expression of *LRA4*, (ii) reduced cell biomass and growth rate, and (iii) improved recombinant protein production (Yan et al. 2018). Additionally, the engineered strain exhibited flocculation and rapid sedimentation at high cell densities (OD_600_ > 6) when it was grown in rhamnose-containing media, particularly YPR medium (Yan et al. 2018).

There are several possible explanations for the different physiological profiles of the engineered strain from the parental strain. Theoretically, down-regulating the expression of *LRA4*, which encodes a rate-limited enzyme involved in rhamnose metabolism, should reduce the utilization efficiency of rhamnose in the engineered strain cultured in rhamnose-containing media (e.g., YPR). In turn, this may result in mild carbon starvation stress due to insufficient rhamnose utilization. Subsequently, numerous physiological profiles including cell viability, autophagy and cell apoptosis, which were reported to be subject to carbon starvation stress (Oda et al. 2015; Weidberg et al. 2011; Wang et al. 2018b), would be altered in the engineered strain to adapt to the mild carbon starvation stress. To verify these speculations and shed light on the related responses, in this study we investigated the differences in transcriptomes and several physiological profiles between the engineered strain *P. pastoris* GS115m/*LacB* and the parental strain *P*. *pastoris* GS115/*LacB* during growth on rhamnose. According to the evidence, we elucidated the molecular mechanism for the improved production of recombinant protein in *P. pastoris* GS115m/*LacB*. Simultaneously, it was disclosed that *P. pastoris* GS115m/*LacB* coped with the mild carbon source starvation mostly via elevating autophagy level. The results would help to understand the survival mechanism responsible for starvation stress including but not limited to carbon source starvation and simultaneously provide a novel strategy for engineering strain to improve produce of target products.

## Materials and methods

### Strains and medium

*Pichia pastoris* GS115m was developed from *P. pastoris* GS115 by replacing the strong rhamnose-inducible promoter P_*LRA4*_ with the weak rhamnose-inducible promoter P_*LRA2*_. In the *P. pastoris* GS115/*LacB* and *P. pastoris* GS115m/*LacB* strains, the β-galactosidase coding gene *LacB* is under the control of the rhamnose-inducible promoter P_*LRA3*_. All strains were described in detail in a previous study (Yan et al. 2018).

The YPD medium contained 1% yeast extract, 2% peptone, and 2% dextrose. The YPR medium contained 1% yeast extract, 2% peptone, and 2% rhamnose. The MR medium contained 1.34% yeast nitrogen base, 4 × 10^−5^% biotin, and 2% rhamnose. To prepare solid medium, agar was supplemented into the above media to a final concentration of 2%.

### Production determination of recombinant protein

Cultivation of *P. pastoris* GS115/*LacB* or *P. pastoris* GS115m/*LacB* in YPR medium and analysis of β-galactosidase activities in the culture supernatants were carried out as previously described (Yan et al. 2018). The wet cell weight (WCW) per milliliter of culture was simultaneously determined at intervals. The β-galactosidase productivity per mg of WCW was assayed based on β-galactosidase activities in the culture supernatants, specific activity of β-galactosidase (575 U/mg), and WCW.

### Total RNA preparation for RNA-seq and real-time polymerase chain reaction (PCR)

Each strain (*P. pastoris* GS115/*LacB* and *P. pastoris* GS115m/*LacB*) was inoculated into YPR medium and grown until an OD_600_ of ~ 2 or ~ 6 was reached. The cells were collected using centrifugation (12,000*g*) at 4 °C for 4 min and stored at − 80 °C before total RNA extraction. Total RNA extraction and trace DNA removal were performed according to previously described methods (Liu et al. 2016). RNA concentration was determined with a Qubit^®^ 2.0 fluorometer (Life Technologies, CA, USA), and the quality was checked using an Agilent RNA 6000 Nano kit combined with an Agilent 2100 Bioanalyzer (Santa Clara, CA, USA).

### RNA sequencing and RNA-seq data analysis

An RNA-seq library was constructed using the NEBNext^®^ Ultra™ RNA Library Prep kit for Illumina^®^ (New England Biolabs, Ipswich, MA, USA) according to the manufacturer’s instructions. RNA library quality confirmation and RNA sequencing were carried out according to the process described by Wang et al. (2018a).

Clean reads were obtained from the raw data by removing low-quality bases (< Q20) using Trim_galore (Bolger et al. 2014). The mapping of clean reads onto the reference genome of *P. pastoris* GS115 was performed using TopHat (v2.0.12) (Trapnell et al. 2009). The strand-specific and unique mapped reads were analyzed using HTSeq (v0.6.1) (Anders et al. 2015). Fragments per kilobase of transcript sequence per millions base pairs sequenced (FPKM) was used to assay the abundance of each gene (Trapnell et al. 2010). Differentially expressed genes (DEGs) were identified using DEseq 2 with an FDR-adjusted *p* value of < 0.05 and the fold-change ≥ 2.

### Real-time PCR

Real-time PCR assays were performed using previously described methods (Yan et al. 2018). The primers used for real-time PCR are listed in Additional file 1: Table S1. The relative expression level of each test gene in *P. pastoris* GS115/*LacB* was assigned as 1, and the glyceraldehyde-3-phosphate dehydrogenase (GAPDH) gene was used as a reference gene. Each gene was analyzed in triplicate, and the data are represented as the means ± (SD) standard deviations.

### Cell viability assay

Cells of *P. pastoris* GS115/*LacB* (WT) and *P. pastoris* GS115m/*LacB* (MT) were grown in liquid YPR medium to an OD_600_ of ~ 6. Cultures were diluted to an OD_600_ of ~ 1 and then washed. After serial dilution (1: 10), 5 μL of samples was spotted on YPR media and 10 μL of samples were spotted on MR media, respectively. The colonies were observed after incubation at 28 °C for 72 h.

### Cell apoptosis analysis

The Annexin V-FITC/PI Cell Apoptosis Detection Kit (TransGen, Beijing, China) was used for Annexin V staining. Cultures of *P. pastoris* GS115/*LacB* and *P. pastoris* GS115m/*LacB* were diluted to an OD_600_ of ~ 0.5 after grown in YPR medium to an OD_600_ of ~ 6, and the cells were washed twice with PBS. Subsequently, the cells were incubated in 100 μL of sorbitol buffer (0.1 M sodium phosphate buffer, 1.2 M sorbitol, pH 7.4) containing 50 U Lyticase (TianGen, Beijing, China) at 28 °C for 30 min. After centrifugation (800*g*, 4 °C, 5 min), cells were washed with 500 μL of 1.2 M sorbitol buffer, and then resuspended in 100 μL of Annexin V binding buffer containing 5 μL of Annexin V-FITC and 5 μL of PI, followed by incubation at room temperature for 15 min. Later, the cells were washed once in 200 μL of Annexin V binding buffer and resuspended in 100 μL of Annexin V binding buffer. Cells were immediately visualized by laser scanning confocal microscope at a 488 nm excitation wavelength, and fluorescence intensities from ~ 20,000 cells were determined by flow cytometry (BD LSRFortessa).

### Staining of autophagosomes

Cell Meter™ Autophagy Assay Kit *Blue Fluorescence* (AAT Bioquest, Sunnyvale, California, USA) was used for autophagosomes staining. *P. pastoris* GS115/*LacB* or *P. pastoris* GS115m/*LacB* was diluted to an OD_600_ of ~ 0.5 when the cells were grown in YPR medium to an OD_600_ of ~ 6. The cells were collected by centrifuge and washed twice with PBS, and then were incubated in 500 μL of stain buffer with 1 μL 500 × autophagy blue™ at 28 °C for 60 min. After incubated the cells were washed twice with 500 μL of washing buffer at 800 g for 5 min at 4 °C, and then resuspended in 100 μL of washing buffer. Cells were immediately visualized by laser scanning confocal microscope.

### Detection of reactive oxygen species (ROS)

Intracellular levels of ROS were measured with ROS Assay Kit (BioDee, Beijing, China). *P. pastoris* GS115/*LacB* and *P. pastoris* GS115m/*LacB* were grown in YPR medium to an OD_600_ of ~ 6. The cells were diluted to OD_600_ ~ 0.5. Collected and washed the cells twice with PBS incubated 30 min at 30 °C with 30 μM of dihydrorhodamine 123 (DHR123). After centrifugation (800*g*, 5 min, 4 °C), the cells were washed with 1 mL of PBS and resuspended in 100 mL of PBS, and then were observed using laser scanning confocal microscope at a 488 nm excitation wavelength and an emission wavelength shifting from green (~ 525 nm), and fluorescence intensities from ~ 20,000 cells were monitored by flow cytometry.

## Results

### Recombinant protein production in *P. pastoris* GS115/*LacB* and *P. pastoris* GS115m/*LacB*

**Fig. 1:**
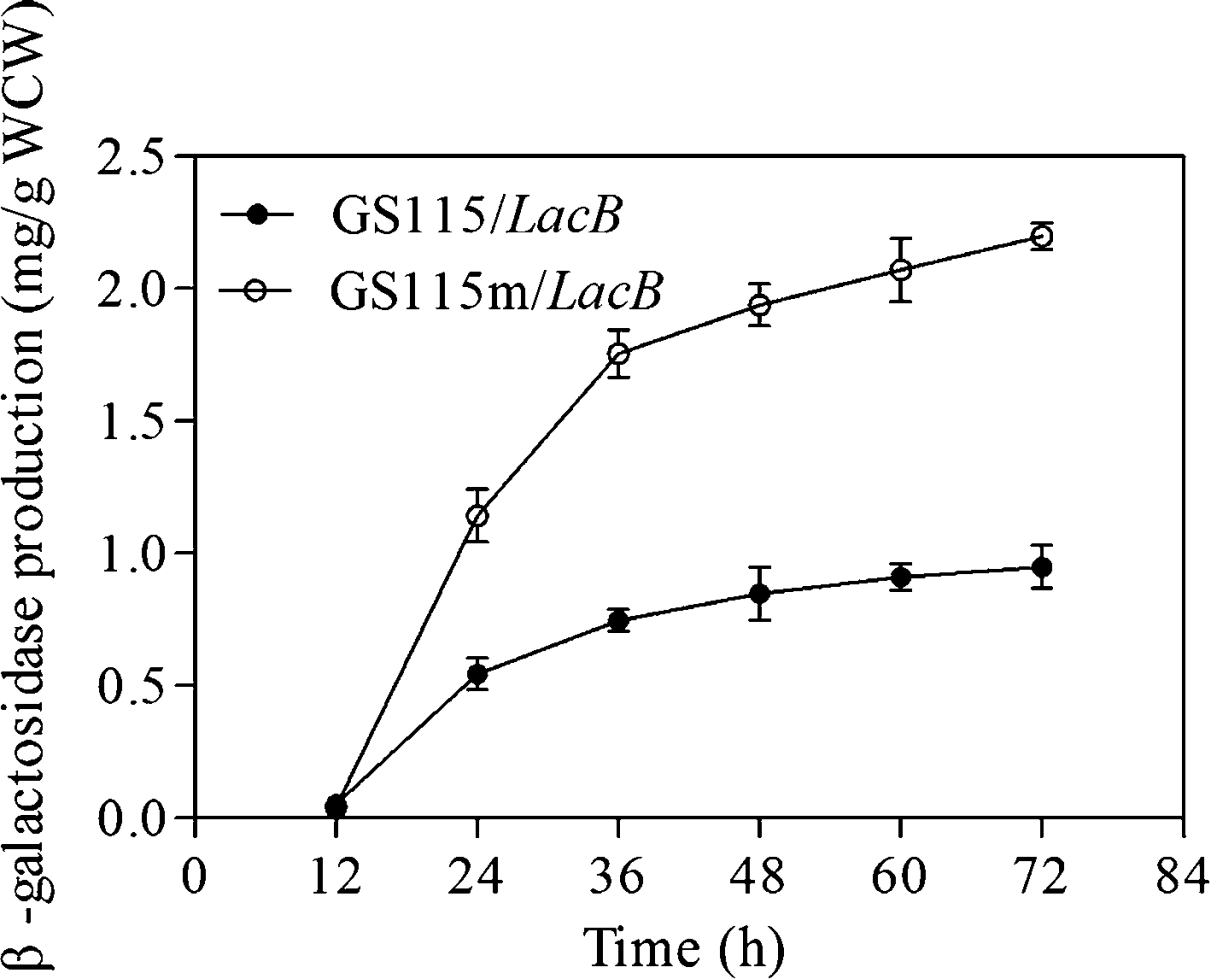
β-Galactosidase production of the test strains cultured in YPR medium. *WCW* wet cell weight

In a previous study, growth rate and maximal biomass were lower for *P. pastoris* GS115m/*LacB* grown in YPR than for *P. pastoris* GS115/*LacB*. However, the amount of recombinant protein, β-galactosidase encoded by *LacB*, in the supernatants of *P. pastoris* GS115m/*LacB* cultures was higher than that of *P. pastoris* GS115/*LacB* (Yan et al. 2018). This indicated that *P. pastoris* GS115m/*LacB* produced the target protein more efficiently. To verify this result, the production of recombinant protein in the two strains grown in YPR medium was investigated based on protein production *vs* wet cell weight (WCW). The results revealed that *Pichia* cells efficiently produced target proteins from 12 to 36 h, and the recombinant protein production at 72 h was more than twofold higher in *P. pastoris* GS115m/*LacB* cell than in *P. pastoris* GS115/*LacB* (Fig. 1). The underlying mechanisms for the improved production of recombinant protein, which could provide some important information for strain engineering, were further investigated.

### Global transcriptional profiles of *P. pastoris* GS115/*LacB* and *P. pastoris* GS115m/*LacB*

*Pichia pastoris* GS115/*LacB* and *P. pastoris* GS115m/*LacB* were cultured in YPR medium until an OD_600_ of ~ 2 or ~ 6, respectively. The cells from two biological replicates of *P. pastoris* GS115/*LacB* and *P. pastoris* GS115m/*LacB* were collected, and total RNA from these cells was prepared for transcriptome analysis using RNA-seq. The RNA-seq data were deposited in the CNGBdb (https://db.cngb.org) with Accession Number CNP0000622 and CNP0000710. The overall expression levels in the two biological replicates of each group were highly similar to each other (R^2^ beyond 0.98) (Additional file 2: Fig. S1). At an OD_600_ of ~ 2, a total of 749 DEGs including 281 down-regulated and 468 up-regulated genes were identified among 5,041 genes, including an exogenous gene, *LacB*. The large number of DEGs indicated that the decrease in rhamnose metabolic flux exerted wide-ranging effects on the transcriptomes of *Pichia* cells and thereby led to various changes in physiological profiles.

**Fig. 2:**
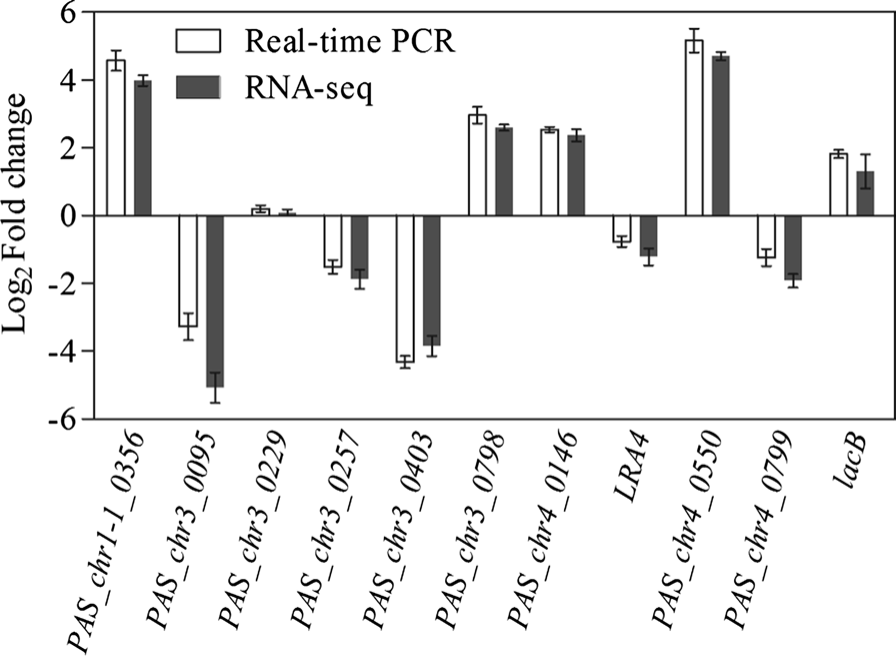
Correlation of RNAseq and real-time PCR. *PAS_chr1*-*1_0356*, hypothetical protein; *PAS_chr3_0095*, phenylpyruvate decarboxylase; *PAS_chr3_0229*, hypothetical protein; *PAS_chr3_0257*, adenylate kinase; *PAS_chr3_0403*, Acetyl-coA synthetase isoform; *PAS_chr3_0798*, hypothetical protein; *PAS_chr4_0146*, hypothetical protein; *LRA4*, L-2-keto-3-deoxyrhamnonate aldolase; *PAS_chr4_0550*, hypothetical protein; *PAS_chr4_0799*, mitochondrial 54S ribosomal protein YmL7/YmL5; *LacB*, β-galactosidase. The corresponding gene in *P. pastoris* GS115/*LacB* was used as the control, and the relative expression value of each gene was designated as 1

To further examine gene expression profiles, real-time PCR was performed to investigate the relative expression of four up-regulated DEGs (*PAS_chr4_0550*, *PAS_chr1*-*1_0356*, *PAS_chr3_0798*, and *PAS_chr4_0146*), four down-regulated DEGs (*PAS_chr3_0095*, *PAS_chr3_0403*, *PAS_chr4_0799*, and *PAS_chr3_0257*), one non-DEG (*PAS_chr3_0229*), and two genes of interest (*LRA4* and *LacB*). The trends in expression of the target genes were consistent between real-time PCR and RNA-seq despite the presence of minor differences in the expression levels of certain genes between the two methods (Fig. 2).

### Improved expression of *LRA3* in *P. pastoris* GS115m/*LacB*

Highly transcribed genes usually play crucial roles in organism survival, and their expression changes under different conditions. According to the FPKM value of each gene, the 25 most highly expressed genes in *P. pastoris* GS115/*LacB* and *P. pastoris* GS115m/*LacB* were identified at an OD_600_ of ~ 2 (Additional file 3: Table S2) and an OD_600_ of ~ 6 (Additional file 4: Table S3), respectively. Theoretically, these genes are expected to play important roles in the survival of *Pichia* cells using rhamnose as the sole carbon source.

Notably, *LRA3* was also one of the 25 most highly expressed genes in both strains grown to OD_600_ ~ 2 and ~ 6, which suggested that *LRA3* expression was intensively induced in the presence of rhamnose. At OD_600_ of ~ 2 and ~ 6, the *LRA3* transcription level was ranked 21st and 14th in *P. pastoris* GS115/*LacB*, and while it was ranked 2nd and 6th in *P. pastoris* GS115m/*LacB*, respectively. These results showed that *LRA3* expression level was significantly elevated in *P. pastoris* GS115m/*LacB* with rhamnose induction. In addition, it was surprising that the *LRA3* transcription level was even higher than that of *GAPDH* in *P. pastoris* GS115m/*LacB*.

### Transcription profiles of the genes related to rhamnose metabolism during incubation

Rhamnose was the main carbon source for cell survival when *Pichia* cells were grown in YPR medium, and the rhamnose utilization rate was therefore a key factor affecting energy production, biomass biogenesis, and physiological profiles in *Pichia* cells. Down-regulating the expression of key rate-determining step enzymes such as LRA4 would decrease rhamnose utilization efficiency, resulting in insufficient supply of energy and carbon matrices for primary and secondary metabolism, growth, and propagation. To adapt to these conditions, the strain should up-regulate the expression of rhamnose-utilization genes to accelerate rhamnose metabolism. This expectation was borne out by transcriptome analysis.

**Fig. 3:**
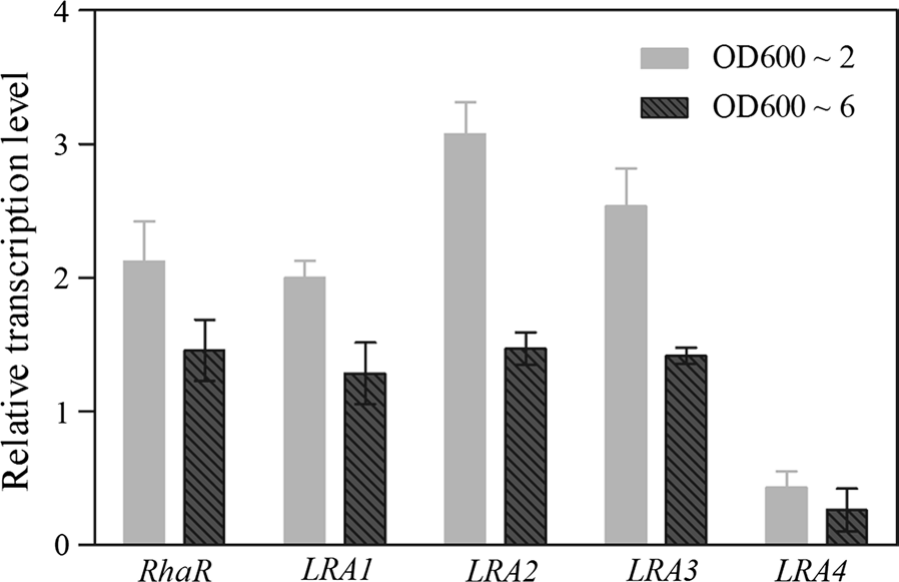
Relative transcription levels of rhamnose utilization genes in *P. pastoris* GS115m/*LacB* grown in YPR medium. Four genes rhamnose utilization of *LRA1*, *LRA2*, *RhaR*, *LRA3*, and *LRA4* was detected in *P. pastoris* GS115m/*LacB* grown in YPR medium to OD_600_ ~ 2 and OD_600_ ~ 6, respectively. The corresponding gene in *P. pastoris* GS115/*LacB* was used as the control, and the relative expression value of each gene was designated as 1

The genes involved in rhamnose metabolism included five genes such as four enzyme-coding genes (*LRA1*–*4*) and a regulator-coding gene (*RhaR*). As expected, the expression levels of all the genes except for *LRA4* in *P. pastoris* GS115m/*LacB* were differentially up-regulated more than twofold compared with *P. pastoris* GS115/*LacB* at an OD_600_ of ~ 2 (Fig. 3). Simultaneously, *LRA4* maintained its low expression as it was under the control of the weak promoter P_*LRA2*_ (Fig. 3). These results indicated that the low production rate of the key rhamnose metabolism-related enzyme LRA4 led to inadequate production of energy and carbon matrices required for normal growth, and *P. pastoris* GS115m/*LacB* therefore enhanced the expression of rhamnose metabolism-related genes to increase rhamnose utilization to provide more energy and biomass for cell growth.

To further investigate the expression profiles of these genes during incubation, the genes were examined when the strains were grown to a high cell density (OD_600_ ~ 6). Relative expression of all genes, except for *LRA4*, decreased compared with that at an OD_600_ of ~ 2 (Fig. 3). When grown to OD_600_ ~ 12, the expression levels of these genes in *P. pastoris* GS115m/*LacB* were almost equal to those of *P. pastoris* GS115/*LacB* (with the exception of *LRA4*), which was reported previously (Yan et al. 2018). This could be explained that the decreasing concentration of residual rhamnose in the medium made a declining induction to the related gene expression. Overall, the relative expression of genes other than *LRA4* was dynamic; it was closely associated with the concentration of residual rhamnose in the medium and decreased with the consumption of rhamnose.

### *LacB* expression profiles during incubation

**Fig. 4:**
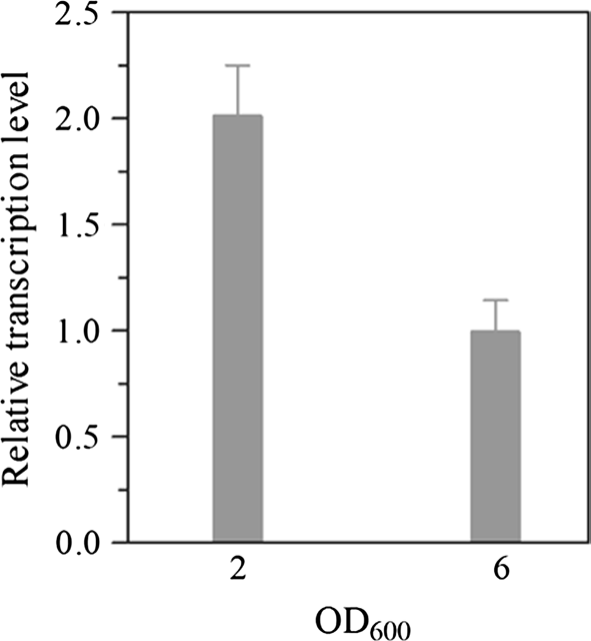
Relative transcription levels of *LacB* in *P. pastoris* GS115m/*LacB* grown in YPR medium. The *P. pastoris* GS115m/*LacB* strain was grown in liquid YPR medium to an OD_600_ of ~ 2 and ~ 6, respectively. The relative expression value of *LacB* in *P. pastoris* GS115/*LacB* was used as the control and designated as 1

Recombinant protein production, an important index for an expression system, was closely and positively dependent on the transcription activity of its promoter. In *P. pastoris* GS115m/*LacB*, *LacB* expression was controlled under P_*LRA3*_, and thereby the production of the recombinant protein, β-galactosidase encoded by *LacB*, was largely subject to the transcriptional activity of P_*LRA3*_. As mentioned above, *LRA3* was one of the most highly transcribed genes, and *LRA3* expression was greatly enhanced in *P. pastoris* GS115m/*LacB* (Additional file 3: Table S2). Under the control of the same promoter, P_*LRA3*_, the trend in *LacB* expression was consistent with that of *LRA3* (Fig. 4). The production of recombinant protein in *P. pastoris* GS115m/*LacB* improved with the increase of P_*LRA3*_ transcription activity.

### Declined cell viability in *P. pastoris* GS115m/*LacB* grown on rhamnose

Generally, rhamnose metabolism was down-regulated due to the decreased expression of the rate-limiting enzyme LRA4. Low rhamnose metabolism was accompanied by low energy supply and reduced sources of carbon-based biomass components. This resulted in decreased growth rate and declined cell biomass in *P. pastoris* GS115m/*LacB* grown in YPR medium, which was confirmed by the results of our previous study (Yan et al. 2018). Additionally, it was reported that cell viability was also affected by carbon starvation such as glucose shortage (Oda et al. 2015).

**Fig. 5:**
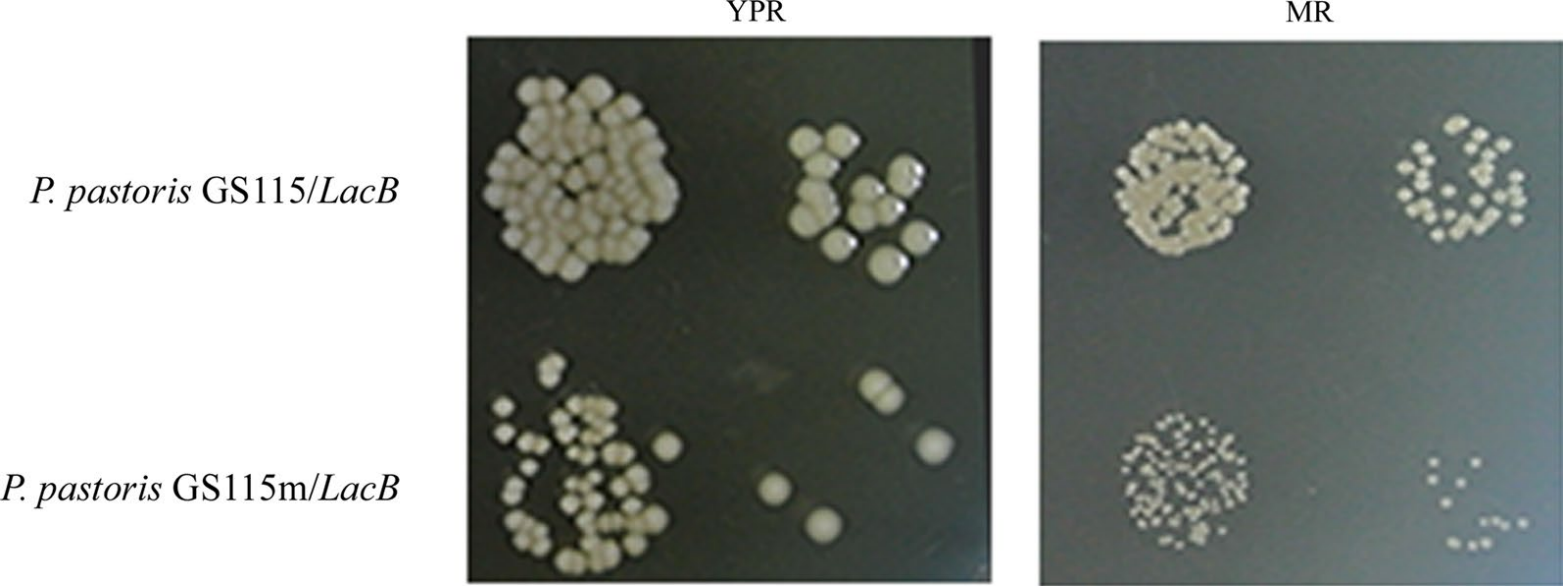
The phenotypes of *P. pastoris* GS115/*LacB* and *P. pastoris* GS115m/*LacB* grown on YPR and MR. The two strains were grown in liquid YPR medium to an OD_600_ of ~ 6, and the cultures were diluted to an OD_600_ of ~ 1, and then serially diluted. After dilution with 10^3^ and 10^4^ times, 5 μL and 10 μL of the diluted cultures were spotted onto YPR and MR, respectively. The colonies were recorded after incubation at 28 °C for 72 h

To confirm whether cell viability of *P. pastoris* GS115m/*LacB* altered, cell growth assay was performed. Differences in the number and size of cell colonies indicated the various profiles of cell viability and generation time of the tested strains, respectively. Small colonies as well as decreased number of cell colonies were observed in *P. pastoris* GS115m/*LacB* compared with *P. pastoris* GS115/*LacB* when rhamnose as the carbon source (YPR and MR) (Fig. 5), which indicated prolonged propagation and declined viability in *P. pastoris* GS115m/*LacB* cells. This led to a lower biomass of *P. pastoris* GS115m/*LacB* grown in YPR and MR, which was described in another study (Yan et al. 2018).

### Increased autophagy level in *P. pastoris* GS115m/*LacB* during growth on rhamnose

Autophagy is a principal catabolic pathway for degrading cellular components including organelles and dysfunctional proteins. Some nonessential cellular components can be degraded via autophagy to synthesize critical components (Devenish and Prescott 2015; Olsvik et al. 2019). Autophagy occurs at low levels under normal conditions (Huang et al. 2015) and increases under adverse conditions such as nutrient deficiency, hypoxia, and oxidative stress (Onodera and Ohsumi 2005; Scherz-Shouval and Elazar 2011; Shpilka et al. 2015; Weidberg et al. 2011). Similarly, autophagy might be induced by the insufficient carbon metabolism due to the decreased rhamnose utilization.

**Fig. 6:**
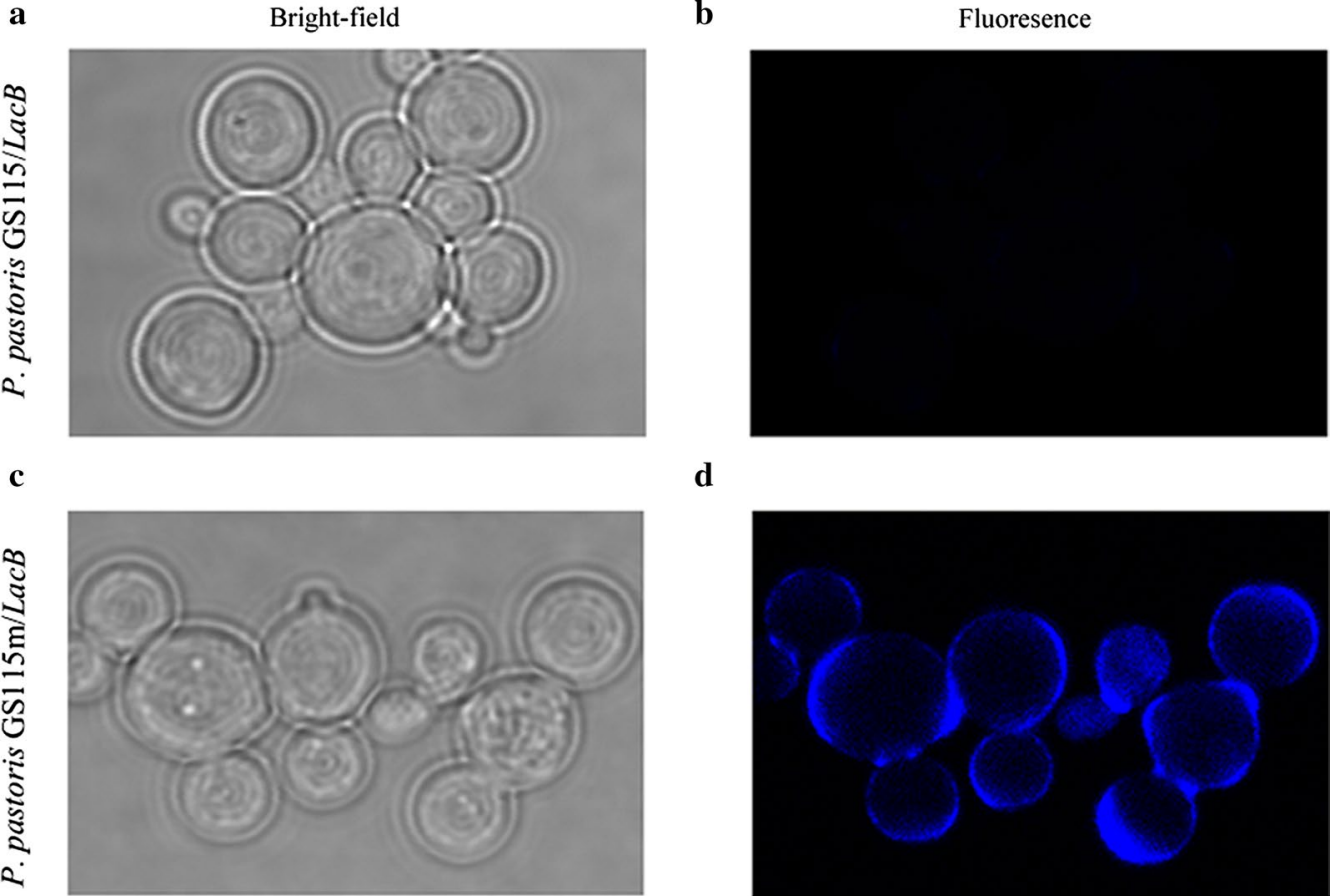
Autophagy profiles in *P. pastoris* GS115/*LacB* and *P. pastoris* GS115m/*LacB* cells. The two strains were grown in YPR to an OD_600_ of ~ 2, and then visualized by laser scanning confocal microscope with a 405 nm excitation wavelength and an emission wavelength shifting from blue (~ 450 nm) after staining with Autophagy Assay Kit

To investigate that autophagy could be triggered by the mild carbon starvation, the autophagy in *P. pastoris* GS115/*LacB* and *P. pastoris* GS115m/*LacB* cells was monitored using autophagosomes staining. Intensive autophagy signals were detected in *P. pastoris* GS115m/*LacB* cells compared with *P. pastoris* GS115/*LacB* cells. Obviously, the carbon starvation arose from the decrease of rhamnose metabolism indeed caused autophagy in *P. pastoris* GS115m/*LacB* (Fig. 6). We assumed that *P. pastoris* GS115m/*LacB* cells recycled non-essential components to synthesize essential components for cell survival and reduce cell apoptosis via autophagy under carbon starvation.

### Undetectable effect on cell apoptosis due to decreased *LRA4* expression

**Fig. 7:**
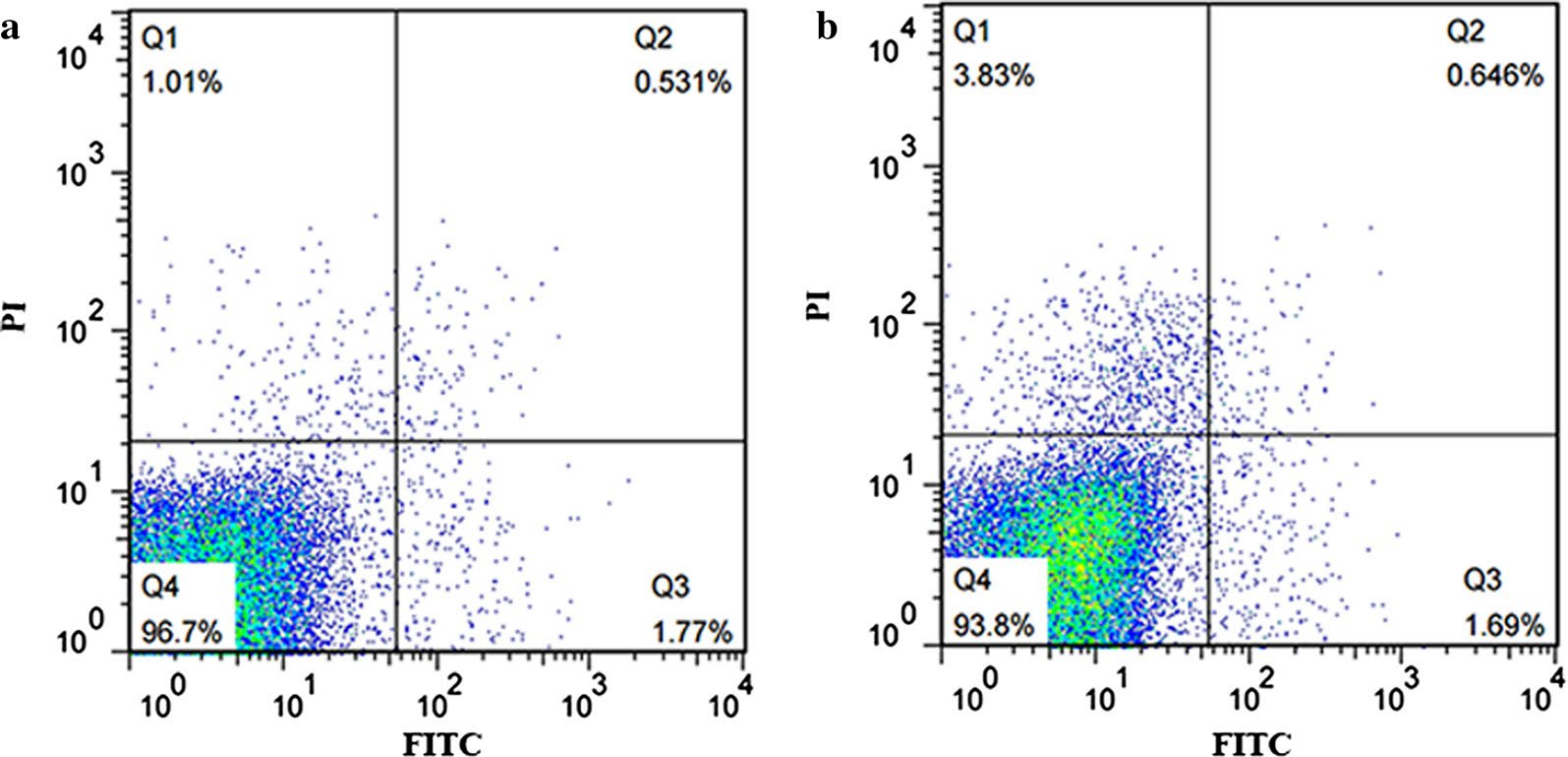
Cell apoptosis profiles of *P. pastoris* GS115/*LacB* (**a**) and *P. pastoris* GS115m/*LacB* (**b**), respectively. Fluorescence intensities in 20,000 cells stained with the Annexin V-FITC/PI apoptosis detection kit were monitored by flow cytometry

Autophagy is interconnected with apoptosis because both of them might be triggered by same signals. Autophagy happened in *P. pastoris* GS115m/*LacB*, and apoptosis was thereby concerned. In order to understand whether apoptosis underwent in *P. pastoris* GS115m/*LacB*, apoptosis profiles in cells of *P. pastoris* GS115m/*LacB* and *P. pastoris* GS115/*LacB* was analyzed by flow cytometry using the Annexin V-FITC/PI apoptosis detection kit. Relatively low apoptosis level occurred in both kinds of cells, and no differences were observed in them (Fig. 7). It indicated that apoptosis did not obviously occur in cells of *P. pastoris* GS115m/*LacB* and *P. pastoris* GS115/*LacB* although the intensive autophagy occurred in *P. pastoris* GS115m/*LacB*. The results showed that the decreased rhamnose metabolism only led to a mild carbon source starvation, which was different from the carbon source starvation due to depletion of carbon source, and brought to a slight alteration of physiological state of *P. pastoris* GS115m/*LacB* instead of death such as apoptosis.

### Low level of reactive oxygen species (ROS) in *P. pastoris* GS115m/*LacB*

**Fig. 8:**
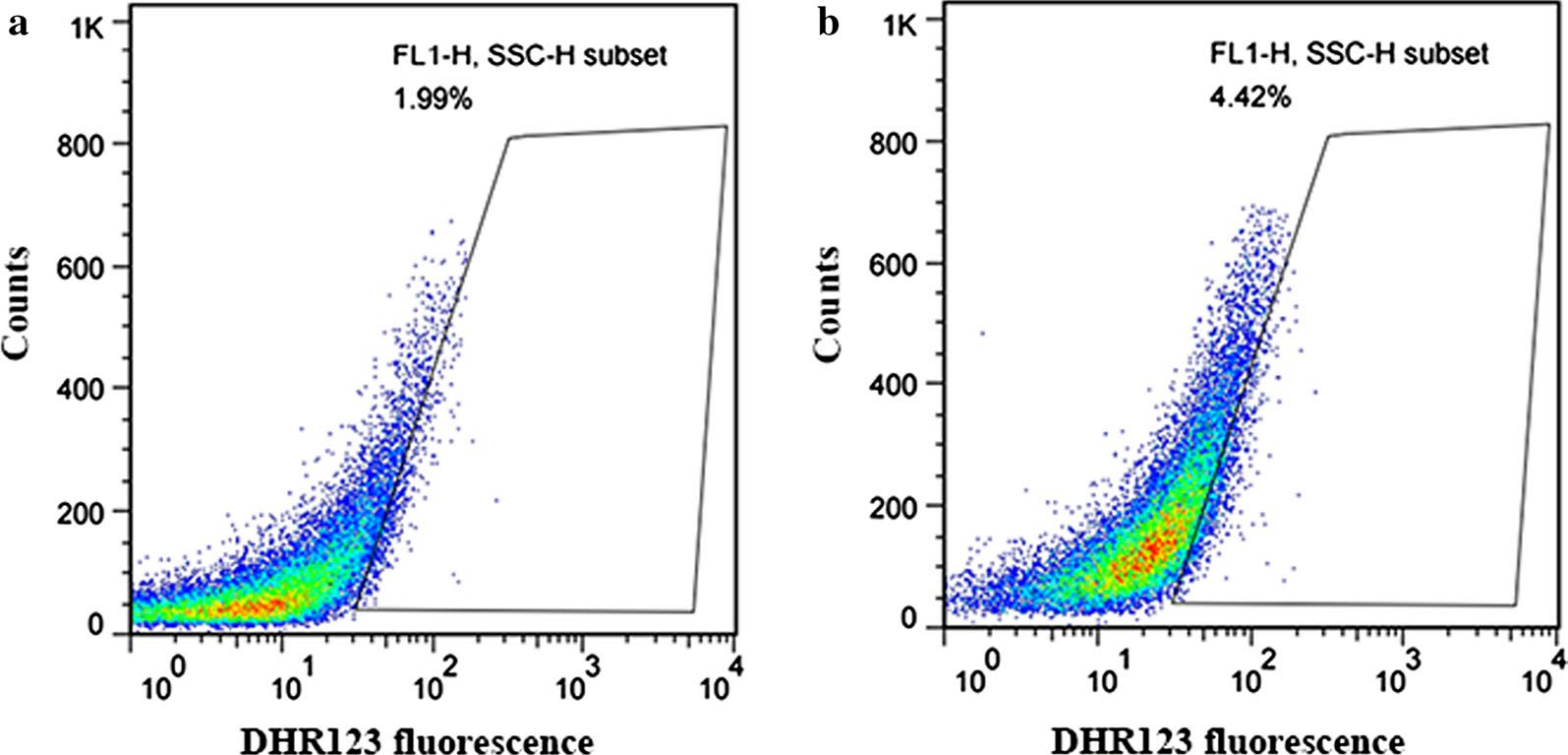
The intracellular ROS in *P. pastoris* GS115/*LacB* (**a**) and *P. pastoris* GS115m/*LacB* (**b**) using DHR-123. Fluorescence intensities in ~ 20,000 cells were monitored by flow cytometer

ROS, which at a high level can induce apoptosis (Sullivan and Chandel 2014), was elevated in production when cells survived nutrient starvation such as inadequate supply of glucose (Wang et al. 2018b). Apoptosis did not occur in *P. pastoris* GS115m/*LacB*, indicating a low level of ROS in *P. pastoris* GS115m/*LacB* cells. The low levels of ROS in both kinds of cells were further confirmed by flow cytometry after DHR123 staining (Fig. 8).

## Discussion

Rational metabolic engineering has been adopted to enhance the production of target products (Maervoet et al. 2016; Song and Lee 2015), such as deleting the bypass pathway or/and strenghening the precursor synthetic pathway (Fan et al. 2016). In the P_*LRA3*_ system, recombinant proteins were the primary products, and production of recombinant proteins was directly related to the transcriptional activity of P_*LRA3*_. P_*LRA3*_ activity was positively correlated to two factors, rhamnose concentration and rhamnose induction duration. We considered that the increase in the two factors could be realized via decreased metabolism, so the engineering strain was developed by down-regulating the expression of one of key rhamnose utilization related genes, *LRA4*. As expected, the engineering strains presented the expected profile, increasing the production of target proteins. However, the actual mechanism on the improved production was not elucidated in detail.

In this study, P_*LRA3*_ activity in *P. pastoris* GS115m/*LacB* as well as *P. pastoris* GS115/*LacB* was disclosed by determining the transcriptional level of *LRA3* in the transcriptome. P_*LRA3*_ was found to be one of the strongest promoters in the presence of rhamnose, and this indicated that recombinant proteins would be highly expressed under the control of P_*LRA3*_. Simultaneously, it was noted that P_*LRA3*_ activity was lower than that of P_*GAP*_ in the parental strain while higher in the engineering strain, suggesting an improved transcriptional level of P_*LRA3*_ in the engineering strain. Transcriptional level of recombinant protein gene, *LacB*, under the control of P_*LRA3*_ was thereby enhanced in the engineering strain, leading to an improved production of LacB. Totally, the elevated P_*LRA3*_ activity directly contributed to the improved production of recombinant proteins.

Rhamnose utilization efficiency in the engineering strain was confirmed to decrease in our previous study. The decreased rhamnose metabolism caused carbon starvation to some extent, and the host would alter some physiological profiles to adapt this kind of stress although this stress was different from the carbon starvation stress arose from glucose depletion. To present, numerous studies have been carried out to investigate carbon starvation due to carbon depletion (Adachi et al. 2017; Marshall and Vierstra 2018; Schwarz et al. 2017), and few reporters have focused specifically on low metabolic flux.

Down-regulated expression of *LRA4* exposed *P. pastoris* GS115m/*LacB* to a slight carbon starvation stress. As a rescue strategy, *P. pastoris* GS115m/*LacB* induced the transcription of rhamnose-utilization genes to increase rhamnose metabolism to attenuate the starvation. In a addition, serious carbon starvation usually brought to some changes of physiological profiles, such as ROS, autophagy, and even apoptosis. Autophagy can block apoptosis to maintain cell survival or induce apoptosis to result in cell death, relying on the nutrient situation. The mild starvation due to insufficient utilization of rhamnose seemed no serious damages to the *P. pastoris* GS115m/*LacB* cells because only obvious autophagy instead of apoptosis occurred. *P. pastoris* GS115m/*LacB* reused some unessential components via autophagy to keep cell viability for survival.

Comprehensively, these findings provided insight into the adaptation mechanisms of microbes under insufficient carbon utilization and some strategies for engineering strain.

## Supplementary information

**Additional file 1: Table S**1. Primers used for real-time PCR.

**Additional file 2: Figure S1**. Comparison of gene expression between two biological replicates in RNA-seq analysis.

**Additional file 3: Table S2.** The 25 most highly expressed genes in *P. pastoris* GS115/*LacB* and *P. pastoris* GS115m/*LacB* (OD_600_ ~ 2).

**Additional file 4: Table S3.** The 25 most highly transcribed genes in *P. pastoris* GS115/*LacB* and *P. pastoris* GS115m/*LacB* (OD_600_ ~ 6).

## Data Availability

The RNA-seq data were deposited in the CNGBdb (https://db.cngb.org) with accession number CNP0000622 and CNP0000710. The authors declare that all data supporting the findings of this study are available from the corresponding authors upon request.
