# Supplementary material for: Decreased expression of LRA4, a key gene involved in rhamnose metabolism, caused up-regulated expression of the genes in this pathway and autophagy in Pichia pastoris
Source: AMB Express. 2020 Feb 25;10:37. doi: 10.1186/s13568-020-00971-2 (PMC7042458; doi:10.1186/s13568-020-00971-2)
Supplement: Supplementary file 1 — Additional file 1: Table S1. Primers used for real-time PCR. [file 13568_2020_971_MOESM1_ESM.docx]

**Table S**1. Primers used for real-time PCR.

| Gene | Sequence (5′ to 3′) |
| --- | --- |
| PAS_chr1-1_0356-F | CCAACCTCATCTCCTCTCTAATCTC |
| PAS_chr1-1_0356-R | ATGGGCGAGTCTACACATCTGA |
| PAS_chr3_0095-F | CTCTCTCTGCCAAGATTGCTGA |
| PAS_chr3_0095-R | CAATCTGTCCTGGTGCATCACTAG |
| PAS_chr3_0229-F | GCCTTTGCCATTCTATTCACTG |
| PAS_chr3_0229-R | TGTGACTGGCTTGCCCTTGT |
| PAS_chr3_0257-F | GGGAAACCGTCCAAAATGAAG |
| PAS_chr3_0257-R | GGAGTTGCCGCCAAGAAGA |
| PAS_chr3_0403-F | CGTAGTCAGGGTAGACAGGAGTTC |
| PAS_chr3_0403-R | CTGGGAGCAGCATTGACAACT |
| PAS_chr3_0798-F | ATAAGGGCAAATGGTTAGAGAGAGT |
| PAS_chr3_0798-R | TAGTCTCGTCTACCCTCAAGGTGA |
| PAS_chr4_0146-F | CAAAGGGCAAACGTATTCCTG |
| PAS_chr4_0146-R | AGAGACAGGGGATAGAAGTGGTAAC |
| *LRA4*-F | CAGCTTGAATCTCCTTGAAGTAGTG |
| *LRA4*-R | TTGGCGTCATTGGAATCAAG |
| PAS_chr4_0550-F | CCTCACTTGCTTTGTTGTAACGA |
| PAS_chr4_0550-R | CAAGCTGCTCATCTCCTCGTAG |
| PAS_chr4_0799-F | GGACAAAAACTGGGACATTTCTCT |
| PAS_chr4_0799-R | CATCCCTGCCACACTACAACTAC |
| *lacB*-F | GACTTTCGGCGGAACAAACT |
| *lacB*-R | GATAGGATGGCGATGCTTTGAC |
| *GAPDH*-F | GTGGTCATCAAACCGGACTCA |
| *GAPDH*-R | CAAGAAGGTCGTCATCACTGCTC |
