# Supplementary material for: Decreased expression of LRA4, a key gene involved in rhamnose metabolism, caused up-regulated expression of the genes in this pathway and autophagy in Pichia pastoris
Source: AMB Express. 2020 Feb 25;10:37. doi: 10.1186/s13568-020-00971-2 (PMC7042458; doi:10.1186/s13568-020-00971-2)
Supplement: Supplementary file 2 — Additional file 2: Figure S1. Comparison of gene expression between two biological replicates in RNA-seq analysis. [file 13568_2020_971_MOESM2_ESM.docx]

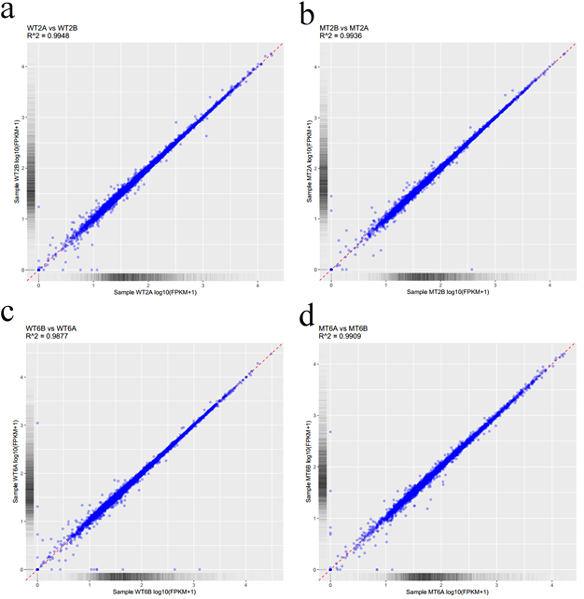


**Figure S1**. Comparison of gene expression between two biological replicates in RNA-seq analysis. **a** WT2A and WT2B, two biological samples of *P. pastoris* GS115/*LacB* grown in YPR until to OD_600_ of ~2; **b** MT2A and MT2B, two biological samples of *P. pastoris* GS115m/*LacB* grown in YPR until to OD_600_ of ~2; **c** WT6A and WT6B, two biological samples of *P. pastoris* GS115/*LacB* grown in YPR until to OD_600_ of ~6; **d** MT2A and MT2B, two biological samples of *P. pastoris* GS115m/*LacB* grown in YPR until to OD_600_ of ~6.
