# Supplementary material for: Decreased expression of LRA4, a key gene involved in rhamnose metabolism, caused up-regulated expression of the genes in this pathway and autophagy in Pichia pastoris
Source: AMB Express. 2020 Feb 25;10:37. doi: 10.1186/s13568-020-00971-2 (PMC7042458; doi:10.1186/s13568-020-00971-2)
Supplement: Supplementary file 3 — Additional file 3: Table S2. The 25 most highly expressed genes in P. pastoris GS115/LacB and P. pastoris GS115m/LacB (OD600 ~ 2). [file 13568_2020_971_MOESM3_ESM.docx]

**Table S2.** The 25 most highly expressed genes in *P. pastoris* GS115/*LacB* and *P. pastoris* GS115m/*LacB* (OD_600_ ~2).

|  | ***P. pastoris* GS115/*LacB*** | | ***P. pastoris* GS115m/*LacB*** | |
| --- | --- | --- | --- | --- |
| Rank | ORF | Product | ORF | Product |
| 1 | PAS_chr4_0407 | Protein of unknown function that associates with ribosomes | PAS_chr1-4_0586 | Hypothetical protein |
| 2 | PAS_chr1-4_0586 | Hypothetical protein | *LRA3* | L-Rhamnonate dehydratase |
| 3 | PAS_chr4_0374 | 60S ribosomal protein L29 | PAS_chr4_0627 | Plasma membrane localized protein that protects membranes from desiccation |
| 4 | PAS_chr2-1_0437 | involved in glycolysis and gluconeogenesis | PAS_chr2-1_0437 | involved in glycolysis and gluconeogenesis |
| 5 | PAS_chr1-4_0504 | 40S ribosomal protein S29 | PAS_chr4_0374 | 60S ribosomal protein L29 |
| 6 | PAS_FragB_0052 | Translational elongation factor EF-1 alpha | PAS_FragB_0052 | Translational elongation factor EF-1 alpha |
| 7 | PAS_chr2-1_0362 | 40S ribosomal protein S26 | PAS_chr4_0407 | Protein of unknown function that associates with ribosomes |
| 8 | PAS_chr1-4_0313 | Subunit 8 of ubiquinol cytochrome-c reductase complex | PAS_chr1-4_0313 | Subunit 8 of ubiquinol cytochrome-c reductase complex |
| 9 | PAS_chr2-1_0429 | One of two nearly identical (see also HTA1) histone H2A subtypes | PAS_chr2-1_0472 | Mitochondrial alcohol dehydrogenase isozyme III |
| 10 | PAS_chr2-2_0265 | hypothetical protein | PAS_chr1-4_0504 | 40S ribosomal protein S29 |
| 11 | PAS_chr4_0348 | 40S ribosomal protein S23 | PAS_chr2-1_0502 | Reduces hydroperoxides to protect against oxidative damage |
| 12 | PAS_chr2-2_0200 | One of two identical histone H4 proteins (see also HHF2) | PAS_chr4_0210 | ADP/ATP carrier protein |
| 13 | PAS_chr2-1_0481 | 40S ribosomal protein S14 | PAS_chr4_0785 | Synthesizes glutamine from glutamate and ammonia |
| 14 | PAS_chr4_0210 | ADP/ATP carrier protein | PAS_chr4_0883 | Hypothetical protein |
| 15 | PAS_chr2-1_0783 | 40S ribosomal protein S18 | PAS_chr2-2_0169 | Hypothetical protein |
| 16 | PAS_chr2-2_0326 | 40S ribosomal protein S25 | PAS_chr2-2_0392 | Outer membrane protein |
| 17 | PAS_chr4_0627 | Plasma membrane localized protein that protects membranes from desiccation | PAS_chr2-2_0265 | hypothetical protein |
| 18 | PAS_chr4_0018 | isoform 1 | PAS_FragB_0061 | Phosphoenolpyruvate carboxykinase |
| 19 | PAS_chr2-1_0472 | Mitochondrial alcohol dehydrogenase isozyme III | PAS_chr2-1_0481 | 40S ribosomal protein S14 |
| 20 | PAS_chr3_1057 | 60S ribosomal protein L32 | PAS_chr3_0837 | Hypothetical protein |
| 21 | *LRA3* | L-Rhamnonate dehydratase | PAS_chr2-2_0326 | 40S ribosomal protein S25 |
| 22 | PAS_chr1-1_0439 | 40S ribosomal protein S8 | PAS_chr4_0786 | Cytosolic superoxide dismutase |
| 23 | PAS_chr2-2_0199 | One of two identical histone H3 proteins (see also HHT2) | PAS_chr4_0348 | 40S ribosomal protein S23 |
| 24 | PAS_chr4_0422 | Subunit VIb of cytochrome c oxidase | PAS_chr2-1_0853 | Hypothetical protein |
| 25 | PAS_chr2-2_0169 | Hypothetical protein | PAS_chr2-1_0783 | 40S ribosomal protein S18 |
