# Supplementary material for: Decreased expression of LRA4, a key gene involved in rhamnose metabolism, caused up-regulated expression of the genes in this pathway and autophagy in Pichia pastoris
Source: AMB Express. 2020 Feb 25;10:37. doi: 10.1186/s13568-020-00971-2 (PMC7042458; doi:10.1186/s13568-020-00971-2)
Supplement: Supplementary file 4 — Additional file 4: Table S3. The 25 most highly transcribed genes in P. pastoris GS115/LacB and P. pastoris GS115m/LacB (OD600 ~ 6). [file 13568_2020_971_MOESM4_ESM.docx]

**Table S3.** The 25 most highly transcribed genes in *P. pastoris* GS115/*LacB* and *P. pastoris* GS115m/*LacB* (OD_600_ ~6).

|  | ***P. pastoris* GS115/*LacB*** | | ***P. pastoris* GS115m/*LacB*** | |
| --- | --- | --- | --- | --- |
| Rank | ORF | Product | ORF | Product |
| 1 | PAS_chr4_0407 | Protein of unknown function that associates with ribosomes | PAS_chr4_0374 | 60S ribosomal protein L29 |
| 2 | PAS_chr1-4_0586 | Hypothetical protein | PAS_chr1-4_0586 | Hypothetical protein |
| 3 | PAS_chr4_0627 | Plasma membrane localized protein that protects membranes from desiccation | PAS_chr4_0407 | Protein of unknown function that associates with ribosomes |
| 4 | PAS_chr2-1_0472 | Mitochondrial alcohol dehydrogenase isozyme III | PAS_chr2-2_0265 | Hypothetical protein |
| 5 | PAS_chr2-2_0265 | Hypothetical protein | PAS_chr1-4_0313 | Subunit 8 of ubiquinol cytochrome-c reductase complex |
| 6 | PAS_chr1-4_0313 | Subunit 8 of ubiquinol cytochrome-c reductase complex | *LRA3* | L-Rhamnonate dehydratase |
| 7 | PAS_chr4_0374 | 60S ribosomal protein L29 | PAS_chr1-4_0504 | 40S ribosomal protein S29 |
| 8 | PAS_chr2-2_0200 | One of two identical histone H4 proteins (see also HHF2) | PAS_chr2-2_0200 | One of two identical histone H4 proteins (see also HHF2) |
| 9 | PAS_chr2-1_0437 | Involved in glycolysis and gluconeogenesis | PAS_chr2-2_0169 | Hypothetical protein |
| 10 | PAS_chr2-1_0429 | One of two nearly identical (see also HTA1) histone H2A subtypes | PAS_FragB_0052 | Translational elongation factor EF-1 alpha |
| 11 | PAS_chr4_0018 | Isoform 1 | PAS_chr2-1_0437 | Involved in glycolysis and gluconeogenesis |
| 12 | PAS_FragB_0052 | Translational elongation factor EF-1 alpha | PAS_chr2-1_0362 | 40S ribosomal protein S26 |
| 13 | PAS_chr1-4_0504 | 40S ribosomal protein S29 | PAS_chr2-1_0429 | One of two nearly identical (see also HTA1) histone H2A subtypes |
| 14 | *LRA3* | L-Rhamnonate dehydratase | PAS_chr4_0348 | 40S ribosomal protein S23 |
| 15 | PAS_chr2-1_0362 | 40S ribosomal protein S26 | PAS_chr4_0018 | Isoform 1 |
| 16 | PAS_chr2-2_0199 | One of two identical histone H3 proteins (see also HHT2) | PAS_chr2-1_0481 | 40S ribosomal protein S14 |
| 17 | PAS_chr2-2_0169 | Hypothetical protein | PAS_chr4_0422 | Subunit VIb of cytochrome c oxidase |
| 18 | PAS_chr4_0422 | Subunit VIb of cytochrome c oxidase | PAS_chr2-1_0472 | Mitochondrial alcohol dehydrogenase isozyme III |
| 19 | PAS_chr1-1_0407 | Hypothetical protein | PAS_chr2-1_0751 | Subunit of the mitochondrial F1F0 ATP synthase |
| 20 | PAS_chr4_0210 | ADP/ATP carrier protein | PAS_chr4_0210 | ADP/ATP carrier protein |
| 21 | PAS_chr4_0348 | 40S ribosomal protein S23 | PAS_chr2-2_0326 | 40S ribosomal protein S25 |
| 22 | PAS_chr1-1_0322 | Subunit 7 of the ubiquinol cytochrome-c reductase complex | PAS_chr2-1_0363 | The terminal member of the mitochondrial inner membrane |
| 23 | PAS_chr2-1_0313 | Hypothetical protein | PAS_chr2-1_0783 | 40S ribosomal protein S18 |
| 24 | PAS_chr2-1_0481 | 40S ribosomal protein S14 | PAS_chr4_0520 | Hypothetical protein |
| 25 | PAS_chr2-1_0363 | The terminal member of the mitochondrial inner membrane | PAS_chr1-1_0107 | NADP(+)-dependent glutamate dehydrogenase |
